# Reduced Functional Bed Capacity Due to Inpatient Boarding Is Associated with Increased Rates of Left Without Being Seen in the Emergency Department

**DOI:** 10.5811/westjem.47312

**Published:** 2025-11-26

**Authors:** Yosef Berlyand, Timmy Lin, Taylor D. Marquis, Jared S. Anderson, Daniel J. Shanin, Alexis C. Lawrence, Frank L. Overly, David B. Curley, Janette Baird, Anthony M. Napoli

**Affiliations:** Department of Emergency Medicine, Warren Alpert School of Medicine, Brown University, Providence, Rhode Island

## Abstract

**Introduction:**

We evaluated the relationship between inpatient boarding, measured as functional bed capacity, and left-without-being-seen (LWBS) rates. Functional bed capacity is defined as the mean percentage of ED beds available for new and existing patients over a 24-hour period.

**Methods:**

We performed quantile regression models examining the association between LWBS and terciles (low, medium, and high) of functional bed capacity, as well as median admit-to-departure times, controlling for other daily operational metrics. We additionally performed an encounter-level analysis to assess the relationship between functional bed capacity at the time of a patient’s arrival and their likelihood of LWBS. Study sites included one academic, one community, and one pediatric ED in a single, urban medical system.

**Results:**

Our study included 373,388 visits. In the adjusted regression at the daily level, low functional bed capacity was associated with an increase of 1.59% in LWBS compared to high functional bed capacity, which represented a 26.5% relative increase (about three patients) compared to median LWBS of 6.0% (*P* < .001). Larger daily census (+ 0.07% for each additional patient, *P* <.001), resulted in two additional patients LWBS for every 15-patient increase in daily census from the median. Additionally, longer length of stay of discharged patients (+ 0.05% for each minute increase, *P* < .001), resulted in two additional patients LWBS for every 20-minute increase in length of stay from the median. Weekdays relative to weekend days were associated with a 1.28% decrease in LWBS (*P* < .001) (approximately three fewer patients who left without being seen relative to the median LWBS of 6.0%). At the encounter level, functional bed capacity in the low and middle tercile was significantly associated with an increased probability of a patient LWBS (91% and 40% increases, respectively, *P* < .001). Of the patients who LWBS, 9.3% were high acuity, 59.5% medium acuity, and 31.2% low acuity.

**Conclusion:**

Functional bed capacity is a new and pragmatic operational metric strongly associated with left-without-being-seen rates and provides an improved way to measure, study, and communicate the impact of inpatient boarding. We propose using functional bed capacity as a metric in future studies of ED operations. Additional studies that incorporate staffing levels to more accurately approximate functional bed capacity and better characterize its true impact on LWBS rates are needed.

## INTRODUCTION

Emergency department (ED) boarding and crowding in the United States has reached crisis levels, resulting in significant negative effects on care quality, morbidity, mortality, patient experience, and operational efficiency.^1^–^7^ Boarding is defined by the American College of Emergency Physicians as the practice of holding patients in the ED after they have been admitted to the hospital. Boarding occurs due to several challenges, including staffing shortages and a lack of inpatient bed availability, and it is a major driver of ED crowding.^1^,^8^ Boarding of inpatients presents a unique challenge as these patients use limited ED beds and staff, severely limiting the ability to care for new patients who arrive. Moreover, boarding patients typically occupy more private care spaces, creating a disproportionate need to care for acute ED patients in hallways and curtained spaces.^9^

As ED crowding has worsened, so has the percentage of patients who leave without being seen (LWBS).^10^ Since 2020, the volume of patients who LWBS has more than doubled, with the 95^th^ percentile of worst-performing hospitals experiencing an increase in LWBS rates from 4.4% in January 2020 to 10.0% in December 2021. The LWBS rates are a core measure (Measure ID OP-22) tracked by the Centers for Medicare & Medicaid Services Hospital Outpatient Quality Reporting Program as a quality metric of ED throughput.^11^ Similar to boarding and crowding, higher LWBS rates correlate with poor patient outcomes, as well as worse patient experience and staff satisfaction^4^,^12^–^15^ and represent lost revenue; therefore, hospital leaders are increasingly interested in curbing rates of LWBS. Many strategies have been proposed to reduce ED crowding and rates of LWBS via flow improvement and decrease in length of stay (LOS). These solutions are largely aimed at improving ED efficiency and patient throughput given available resources.

We suspect that one independent driver of rising rates of LWBS is inpatient boarding within the ED. We aimed to study the impact of inpatient boarding, which is primarily a function of hospital operations rather than ED operations,^1^ on the rates of LWBS across multiple EDs within an academic medical system. Emergency department boarding has previously been studied by evaluating boarding times, often measured as admission-to-departure time, or boarder burden, typically defined as the average number of boarding patients per hour.^5^ These metrics are fundamentally tied to specific ED size, are not generalizable across EDs, and do not account for other factors such as staffing limitations. Moreover, they are not intuitive for hospital leaders to understand. We chose to measure boarding as a new variable termed functional bed capacity, defined as the mean percentage of ED beds available for new and existing ED patients over a 24-hour period. We hypothesized that functional bed capacity can more intuitively and accurately capture the impact of boarding and could serve as a pragmatic and more generalizable metric in the field of ED operations.

**Population Health Research Capsule**

What do we already know about this issue?

*Inpatient boarding in the emergency department has reached crisis levels and may be an independent driver of increasing rates of leaving without being seen (LWBS).*

What was the research question?

*Using a novel variable of functional bed capacity, we evaluated the association between inpatient boarding and LWBS rates.*

What was the major finding of the study?

*Low functional bed capacity is associated with an absolute increase of 1.59% in LWBS (P <.001), which is a 26.5% relative increase from the median.*

How does this improve population health?

*The association between inpatient boarding and LWBS highlights another reason to curtail inpatient boarding. Functional bed capacity is a pragmatic metric to track LWBS rates*,

## METHODS

This study was evaluated by our Institutional Review Board (IRB) and deemed exempt from IRB approval. Using a retrospective observational study design, we evaluated patient encounters between October 1, 2022–June 11, 2024 across three EDs within an academic medical system consisting of a large, academic, urban, adult ED (site A); a medium-sized, urban community ED (site B); and an academic, urban. pediatric ED (site C). We collected data from an already existing dataset of metrics without the need for individual chart review. Site A is a 706-bed tertiary care academic medical center with an annual ED patient volume of 89,406, 75 ED beds, and has Level I trauma, ST-elevation myocardial infarction (STEMI)-receiving, and comprehensive stroke center designations. Site B is a 247-bed community hospital with an annual ED patient volume of 74,239, 47 ED beds, and has STEMI-receiving and primary stroke center designations. Site C is an 87-bed pediatric, academic medical center with an annual ED patient volume of 53,772, 41 ED beds, with Level I pediatric trauma and primary stroke center designations. Our study included a total of 373,388 visits across the three hospitals, broken down as 152,166, 126,235, and 94,987 at sites A, B, and C, respectively.

We assessed daily operational metrics using an existing quality improvement (QI) dataset that includes arrivals per hour, daily arrival volume (census), number of patients who left without being seen (LWBS total), percentage of census that left without being seen (LWBS percentage), median ED length of stay (LOS), median LOS of discharged patients (LOSD), median LOS of admitted patients (LOSA), and number of boarding patients at the top of the hour every hour (hourly boarder burden). At the encounter level, we collected age, arrival method, and patient acuity as indicated by the Emergency Severity Index (ESI); ESI 4 and 5 are categorized as low acuity, ESI 3 as medium acuity, and ESI 1 and 2 as high acuity.

To allow for interpretation not contextualized by ED size, we transformed hourly boarder burden into a new variable termed functional bed capacity. We calculated an estimated functional bed capacity by taking the difference between the number of licensed ED beds and the average number of boarding inpatients at the top of each hour as a percentage of licensed ED beds. As an example, if the average hourly boarder burden is 13 patients in an ED with 75 beds, then the functional. bed capacity is 82.6%. This linear transformation allows for interpretation of the impact of boarding irrespective of ED size.

**Figure 1:**
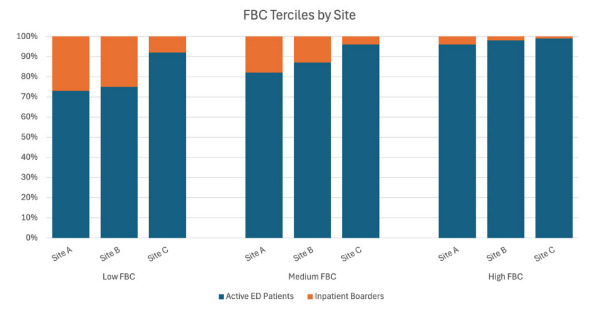
Terciles of functional bed capacity by site in a study to determine whether this operational metric is correlated with rates of left without being seen in the emergency department. *ED*, emergency department; *FBC*, functional bed capacity.

Given the distribution of the outcome (LWBS) and time variables (admission to departure), we performed unadjusted and adjusted quantile regression models examining the association between LWBS and the tercile of functional bed capacity, as well as median admission-to-departure times for comparison, adjusting for other covariates. We performed a similar encounter-level analysis to assess the relationship between functional bed capacity at the time of a patient’s arrival and the likelihood that the patient will LWBS, adjusting for other covariates. Quantile regression is a more robust analysis for data skewed by extreme outliers and is not limited by the assumptions of the parametric distribution of outcome or predictor variables.^16^,^17^ We selected terciles for ease of interpretation and comparison. These calculations were performed in aggregate across all three sites, as well as individually at each site. For each site, we categorized functional bed capacity as low (first tercile), medium (second tercile), or high (third tercile) (Figure 1). All statistics were performed in SAS v9.4 (SAS Institute, Cary, NC). The unadjusted and adjusted quantile regression model results are reported for the 50^th^ percentile.

## RESULTS

Daily operational metrics are summarized in Table 1. Key metrics include an aggregate median LWBS percentage of 6.02% with an IQR of 2.84–10.18%; median functional bed capacity of 85.21% (IQR 74.97–93.29%); and median LOSD of 331 minutes (IQR 259–388).

### Analysis at the Daily Level

In the unadjusted aggregate model, LWBS percentage was associated with an absolute increase of 5.22% when functional bed capacity is in the lowest tercile (*P* <.001, Table 2), representing an 87.0% relative increase (approximately 10 patients) in comparison to the median LWBS. This association remains significant, albeit reduced, when controlling for daily census, LOSD, season, and weekends in the adjusted model (*P* < 0.001, Table 2). In this model, LWBS is associated with an absolute increase of 1.59% when the functional bed capacity is in the lowest tercile, which represents a 26.5% relative increase (about three patients) compared to the median LWBS rate.

Other significant factors associated with increased LWBS rate include larger daily census (0.07% for each additional patient, *P* < .001, Table 2), longer LOSD (0.05% for each minute increase, *P* < .001, Table 2), and season of fall (+1.64%, *P* < .001, Table 2) and summer (+0.87%, *P* = .001, Table 2), both relative to winter. Every 15-patient increase in daily additional patients from the median resulted in two additional patients LWBS. Every 20-minute increase in LOSD from the median resulted in two additional patients LWBS. Approximately three additional patients left without being seen per day during the fall, and approximately two additional patients LWBS per day during the summer relative to winter. Weekdays compared to weekend days were significantly associated with a 1.28% decrease (approximately three fewer patients who LWBS relative to the median LWBS of 6.0%) in LWBS (*P* <0.001, Table 2). When comparing functional bed capacity to operational variables such as daily census and LOSD, functional bed capacity has the largest impact (Table 2). Similar results were seen within each site, as shown in Table 3.

We performed a similar analysis using median admission-to-departure in place of functional bed capacity, as admission to departure has frequently been cited in boarding literature as an operational metric (Table 4). Substituting median admission to departure as a continuous variable in place of functional bed capacity yielded comparable model performance (Adj-R 0.35 vs 0.35, Tables 4 and 2) with a smaller effect size (one additional patient LWBS for every 100-minute increase in median admission to departure, *P* < .001, Table 4).

### Analysis at the Encounter Level

To better understand the impact of functional bed capacity on an individual patient at the time of their arrival, we performed a patient-encounter level analysis examining the association between functional bed capacity at the time of ED arrival and the probability that an individual will LWBS. This model controlled for patient age, arrivals per hour at the time of the index patient’s arrival, site, acuity, arrival method, season, and day of week. In this model, functional bed capacity in its lowest tercile was associated with a 91% increase in the probability of a patient LWBS (*P* < .001, Table 5), and a 40% increase with functional bed capacity in its middle tercile (*P* < .001, Table 5), compared to functional bed capacity in its highest tercile. Other factors associated with an increased likelihood of LWBS include low acuity with a 534% increase (*P* < .001, Table 5) and medium acuity with a 394% increase (*P* < .001, Table 5), season of fall with a 34% increase (*P* < .001, Table 5) and summer with a 14% increase (*P* < .001, Table 5) relative to winter, and weekday with a 14% increase (*P* < .001, Table 5) relative to weekend. Arrival by emergency medical services (EMS), season of spring relative to winter, and older age were significantly associated with decreased likelihood of LWBS (*P* < .001). Patients arriving by EMS were 39% less likely to LWBS. Patients presenting to the ED in the spring compared to winter were 19% less likely to LWBS. There was a 2% reduction in the likelihood of LWBS for each unit increase in age from the site-specific median age. Of the patients who LWBS, 9.3% were high acuity, 59.5% medium acuity, and 31.2% low acuity.

## DISCUSSION

In this retrospective, multisite, observational cohort study, we demonstrated that LWBS percentage increases with decreasing ED bed availability due to boarding of inpatients, as measured by functional bed capacity. This association holds true after controlling for other factors thought to influence LWBS, including day of week, season, LOSD patients, and ED arrivals. This is the first study to demonstrate a relationship between functional bed capacity and rates of LWBS, and our findings were replicable across an academic medical center, a community hospital, and a pediatric ED. After controlling for confounders, low functional bed capacity was associated with a 1.59% absolute rise in LWBS and a 26.5% increase relative to median LWBS. At the encounter level, functional bed capacity in its lowest tercile or middle tercile at the time of patient arrival was associated with an increased risk of LWBS, even after controlling confounding variables.

At the daily level, we found that low functional bed capacity was consistently associated with increased LWBS rates. At the encounter level, however, both middle and low functional bed capacity at the time of a patient’s arrival increased their odds of LWBS. This result was likely seen due to the averaging of our daily analysis over a 24-hour period. Even when functional bed capacity was in the middle tercile at the time of patient arrival, patients were statistically more likely to LWBS.

At the daily level, we found that LWBS rates were higher over the weekend relative to weekdays, and higher in fall relative to winter. The day-of-week association is the reverse of what was seen in the encounter-level analysis, likely because patients were analyzed by the date of arrival rather than date of LWBS. As an example, a patient who arrived on a Friday evening and LWBS on Saturday after midnight would be considered a weekend LWBS on the daily analysis but a weekday LWBS at the encounter. level. Although we were unable to account for staffing in our model, we suspect that weekend LWBS rates are driven, in part, by reduced staffing throughout the ED and hospital, which likely reduces available ED bed space and worsens throughput metrics. Patient-specific factors, such as ability or desire to wait to be seen, may also play a role in this observation. Strikingly, 68.8% of patients who LWBS during the period studied were of high or moderate acuity. This highlights the extraordinary patient safety concern of patients who LWBS.

While prior studies aiming to explain LWBS focused on other measures of ED throughput, most were performed prior to the nationwide boarding crisis currently impacting EDs. Today, EDs are tasked with improving throughput in the face of fewer care spaces and greater staffing shortages, combined with rising patient arrivals and acuity. To reduce LOS and LWBS, innovations in ED operations that have been shown to increase productivity and efficiency should be implemented whenever possible, although some commonly implemented strategies have failed to fully mitigate the effects of boarding.^18^ Ultimately, efforts to increase functional bed capacity by curtailing inpatient boarding must be implemented to further drive down LWBS rates. Emergency department crowding and boarding have been studied extensively, and numerous underlying causes have been identified. The Association of Academic Chairs of Emergency Medicine summarized the problem: “The cause of ED crowding is misaligned health care economics that pressures hospitals to maintain inefficient high inpatient census levels… [and] few efforts address the economically driven root causes of ED crowding.”^1^ While ED leaders must innovate in the face of staggering constraints to provide better care to ED patients and reduce LWBS, the solution will necessitate a commitment from hospital leaders to invest in hospital operations.

## LIMITATIONS

Our study has several limitations. First, we were unable to account for ED or inpatient physician and nursing staffing or delays by consulting services, as these data were not available to us. We performed our analysis conservatively and assumed that all ED beds were always staffed; thus, we were likely underestimating the impact of boarding in this analysis. An ideal measure of functional bed capacity would account only for open, staffed beds, which would more accurately reflect the average percentage of ED beds available to care for acute ED patients over a 24-hour period. Second, as we conducted. a retrospective study with limited access to clinical data, we were unable to account for different clinical presentations, detailed demographics, or other patient-specific factors aside from acuity, which may have resulted in unmeasured confounders. We were also unable to assess the clinical outcomes or social determinants of health of patients who LWBS nor to account for factors related to other services outside the ED. Third, our baseline rates of LWBS were not representative of the national median, and our study was performed at urban hospitals, which may limit the generalizability of our findings.

## CONCLUSION

This retrospective, observational, multisite study shows that the percentage of patients who left without being seen was independently driven by boarding inpatient volumes in the ED, as measured by functional bed capacity and by admission-to-departure times. Functional bed capacity has a larger effect than the traditional measure of admission-to departure times and can be translated as a metric across hospitals regardless of ED size.

Functional bed capacity is a new and pragmatic operational metric strongly associated with LWBS and provides an improved way to measure, study, and communicate the impact of inpatient boarding on the ED. Functional bed capacity provides an intuitive framing to the boarding crisis in a way that hospital leaders can easily understand. We propose using functional bed capacity as a metric in future studies of ED operations. Combatting rising LWBS rates must include efforts to increase functional bed capacity, which is directly related to inpatient boarding and staffing levels and requires hospital-level commitment for improvements to occur. Future studies that incorporate staffing levels to more accurately approximate functional bed capacity and better characterize its true impact on LWBS rates are needed, as is research to better characterize the clinical outcomes of patients who leave without being seen and the economic consequences of losing these encounters.

## Figures and Tables

**Table 1:** Daily operational metric summary statistics.

Metric	Aggregate Median (IQR)	Site A Median (IQR)	Site B Median (IQR)	Site C Median (IQR)
Left-without-being-seen percentage	6.0 (2.8, 10.2)	8.9 (5.8, 12.9)	6.02 (3.0, 9.5)	3.3 (1.4, 6.5)
Length-of-stay of discharged patients in minutes	330 (259, 388)	379 (342, 422)	352 (308, 396)	232 (203, 267)
Functional bed capacity	85.2 (75.0, 93.3)	77.6 (71.6, 84.0)	81.3 (71.6, 89.9)	94.7 (91.2, 96.7)
Daily Census	204 (165, 233)	246 (230, 262)	206 (191, 218)	151 (137, 168)

**Table 2:** Unadjusted and adjusted quantile regression models examining the association between percentage of patients who left without being seen and functional bed capacity in aggregate across three study sites at the daily level.

Model	Covariates	Coefficient estimate	P-value	Adj-R
Unadjusted	Functional Bed Capacity (%)			0.088
	Low Tercile	5.22	**< .001**	
	Medium Tercile	2.06	**< .001**	
	High Tercile	-	-	
Adjusted	Functional Bed Capacity (%)			0.350
	Low Tercile	1.59	**< .001**	
	Medium Tercile	0.17	.44	
	High Tercile	-	-	
	Median Daily Census	0.07	**< .001**	
	Median Length of Stay of Discharged Patients	0.05	**< .001**	
	Season			
	Fall	1.64	**< .001**	
	Spring	−0.47	.06	
	Summer	0.87	**.001**	
	Winter	-	-	
	Day Type			
	Weekday	−1.28	**< .001**	
	Weekend	-	-	

Note: Significant *P*-values are highlighted in bold.

*ADJ-R*, pseudo adjusted r-squared.

**Table 3:** Unadjusted and adjusted quantile regression models examining the association between percentage of patients who left without being seen by functional bed capacity by site at the daily level.

	Model	Covariates	Coefficient Estimate	P-value	Adj-R
Site A	Unadjusted	Functional Bed Capacity (%)			0.035
		Low Tercile (50.17–73.49%)	3.66	**< .001**	
		Medium Tercile (73.50–81.60%)	1.11	.13	
		High Tercile (81.61–100%)	-	-	
	Adjusted	Functional Bed Capacity (%)			0.420
		Low Tercile (50.17–73.49%)	1.23	**< .001**	
		Medium Tercile (73.50–81.60%)	0.29	.38	
		High Tercile (81.61–100%)	-	-	
		Median Daily Census	0.06	**< .001**	
		Median Length of Stay of Discharged Patients	0.05	**< .001**	
		Season			
		Fall	2.40	**< .001**	
		Spring	−1.70	**< .001**	
		Summer	−0.11	.82	
		Winter	-	-	
		Day Type			
		Weekday	−1.93	**< .001**	
		Weekend	-	-	
Site B	Unadjusted	Functional Bed Capacity (%)			0.140
		Low Tercile (41.05–75.26%)	5.29	**< .001**	
		Medium Tercile (75.27–86.96%)	2.69	**< .001**	
		High Tercile (86.97–100%)	-	-	
	Adjusted	Functional Bed Capacity (%)			0.490
		Low Tercile (41.05–75.26%)	2.06	**< .001**	
		Medium Tercile (75.27–86.96%)	0.54	.05	
		High Tercile (86.97–100%)	-	-	
		Median Daily Census	0.074	**< .001**	
		Median Length of Stay of Discharged Patient	0.043	**< .001**	
		Season			
		Fall	0.95	**.002**	
		Spring	−0.37	.15	
		Summer	1.26	**.003**	
		Winter	-	-	
		Day Type			
		Weekday	1.21	**< .001**	
		Weekend	-	-	
Site C	Unadjusted	Functional Bed Capacity (%)			0.097
		Low Tercile (55.49–92.37%)	4.76	**< .001**	
		Medium Tercile (92.38–96.13%)	1.53	**< .001**	
		High Tercile (96.14–100%)	-	-	
	Adjusted	Functional Bed Capacity (%)			0.410
		Low Tercile (55.49–92.37%)	1.25	**< .001**	
		Medium Tercile (92.38–96.13%)	−0.31	**.02**	
		High Tercile (96.14–100%)	-	-	
		Median Daily Census	0.065	**< .001**	
		Median Length of Stay of Discharged Patients	0.054	**< .001**	
		Season			
		Fall	0.054	0.20	
		Spring	−0.11	0.39	
		Summer	−0.76	**< .001**	
		Winter	-	-	
		Day Type			
		Weekday	−0.76	**< .001**	
		Weekend	-	-	

Note: Significant *P*-values are highlighted in bold.

*ADJ-R*, pseudo adjusted r-squared.

**Table 4:** Unadjusted and adjusted quantile regression models examining the association between percentage left without being seen by admission-to-departure time in aggregate across the three study sites at the daily level.

Model	Covariates	Coefficient Estimate	P-value	Adj-R
Unadjusted	Admission-to-departure time in minutes	0.015	**< .001**	0.067
Fully Adjusted	Admission-to-departure time in minutes	0.005	**< .001**	0.35
	Median Daily Census	0.071	**< .001**	
	Median Length of Stay of Discharged Patients	0.049	**< .001**	
	Season			
	Fall	1.480	**< .001**	
	Spring	−0.340	.18	
	Summer	0.780	**.01**	
	Winter	-	-	
	Day Type			
	Weekday	−1.260	**< .001**	
	Weekend	-	-	

Note: Significant *P*-values are highlighted in bold.

*ADJ-R*, pseudo adjusted r-squared.

**Table 5:** Encounter-level adjusted quantile regression model examining the probability of leaving without being seen based on functional bed capacity at the time of patient arrival in aggregate across the three study sites.

Covariates	Odds Ratio	P-value	Area under ROCC
Age in years	0.98	**< .001**	0.79
Arrivals per hour	0.98	**< .001**	
Functional Bed Capacity (%)			
Low Tercile	1.91	**<. 001**	
Medium Tercile	1.40	**< .001**	
High Tercile (reference)	-	-	
Site			
A	2.16	**< .001**	
B (reference)	-	-	
C	0.44	**< .001**	
Acuity			
Low Acuity	6.34	**< .001**	
Medium Acuity	4.94	**< .001**	
High Acuity (reference)	-	-	
Arrival by EMS	0.61	**< .001**	
Season			
Fall	1.34	**< .001**	
Spring	0.81	**< .001**	
Summer	1.14	**< .001**	
Winter (reference)	-	-	
Day Type			
Weekday	1.14	**< .001**	
Weeken d (reference)	-	-	

Note: Significant *P*-values are highlighted in **bold**,

*EMS*, emergency medical services; *ROCC*, receiver operating characteristic curve.
